# Interventricular Septal Thickness as a Diagnostic Marker of Fetal Macrosomia

**DOI:** 10.3390/jcm10050949

**Published:** 2021-03-01

**Authors:** Bartosz Szmyd, Małgorzata Biedrzycka, Filip Franciszek Karuga, Magdalena Rogut, Iwona Strzelecka, Maria Respondek-Liberska

**Affiliations:** 1 Department of Pediatrics, Oncology, and Hematology, Medical University of Lodz, 91-738 Łódź, Poland; bartoszmyd@gmail.com; 2 Student’s Scientific Association Prenatal Cardiology, Medical University of Lodz, 93-338 Łódź, Poland; malgorzata.biedrzycka@stud.umed.lodz.pl (M.B.); filipfranciszek439@gmail.com (F.F.K.); magdalena.rogut@stud.umed.lodz.pl (M.R.); 3 Department of Diagnosis and Prevention Fetal Malformations, Medical University of Lodz, 93-338 Łódź, Poland; maria.respondek-liberska@uni.lodz.pl; 4 Department of Prenatal Cardiology, Polish Mother’s Memorial Hospital, 93-338 Łódź, Poland

**Keywords:** fetal macrosomia, interventricular septal thickness, prenatal cardiology

## Abstract

Serious complications in both mother and newborn arising as a result of fetal macrosomia indicate the need for early diagnosis and prevention. Unfortunately, current predictors, such as fetal biometry, fundal height, and amniotic fluid index, appear to be insufficient. Therefore, we decided to assess the predictive potential of interventricular septal thickness (IVST), as measured at ≥33 weeks of gestation. Two hundred and ninety-nine patients met the inclusion criteria: complete medical history including all necessary measurements—namely, IVST obtained by M-mode echocardiography, fetal biometry, and birth weight. The Statistica 13.1 PL software was used to generate the receiver operating curve. The optimal cut-off point (IVST of 4.7 mm) was selected using the Youden index method. The analysis of fetal biometry abnormalities resulted in 46.6% of macrosomia cases being correctly predicted; however, IVST analysis detected 71.4% of cases. IVST at ≥4.7 mm appears to have a higher sensitivity and negative predictive value (NPV) than routine ultrasound.

## 1. Introduction

Intuitively, *fetal macrosomia* refers to a fetus or newborn which is much larger than average. The American College of Obstetricians and Gynecologists (ACOG) defines it as an absolute birth weight of over 4000 g, regardless of gestational age [1]. The impact of gestational age on excessive fetal growth is reflected in another term, *large for gestational age* (LGA), which pertains to fetuses with estimated fetal weight (EFW) at ≥90th percentile for a given gestational age [1].

**Figure 1:**
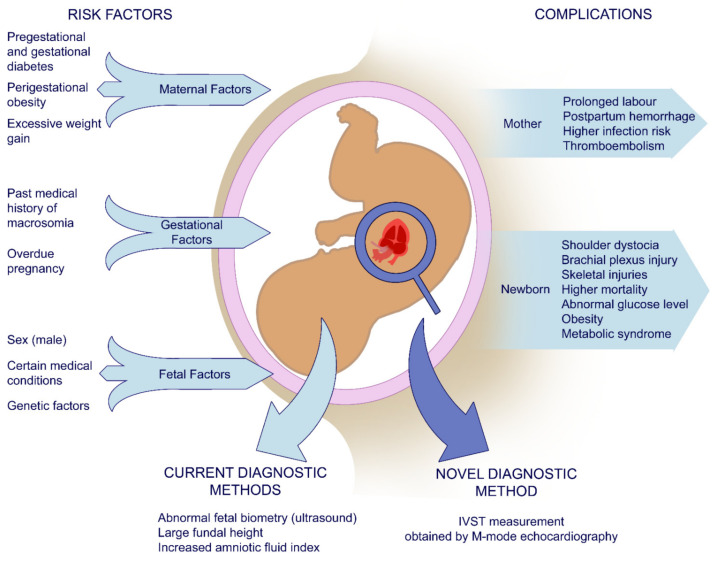
Schematic representation of maternal, gestational, and fetal risk factors of macrosomia and related complications and parameters used in the diagnostic process. IVST—interventricular septal thickness.

Factors predisposing to macrosomia can be divided into three groups: maternal, gestational, and fetal. Among the main maternal risk factors are pre-gestational or gestational diabetes and a positive history of macrosomia in previous pregnancies [2,3,4,5]. As shown in Figure 1, fetal factors include gender, genetics, and certain medical conditions [1,6]. Moreover, high paternal BMI might also play a role [7].

Serious complications in both mother and newborn (Figure 1) arising as a result of macrosomia indicate the need for early diagnosis and prevention [1]. Unfortunately, current predictors such as fetal biometry, fundal height, and amniotic fluid index appear to be insufficient [8,9]. This has been humorously described in the latest ACOG Practice Bulletin: “*Parous women seem to be able to predict the weight of their newborns as well as clinicians who use ultrasonography or clinical palpation maneuvers*” [1].

Based on our experience, we have selected the interventricular septal thickness (IVST) as a potential diagnostic marker of fetal macrosomia. Changes in IVST after 33 weeks of gestation are minimal, and therefore this cut-off point was chosen as the most appropriate [10,11]. Therefore, we decided to assess the diagnostic potential of interventricular septal thickness, as measured at ≥33 weeks of gestation.

## 2. Materials and Methods

### 2.1. Study Design and Participants

We retrospectively analyzed the medical records of fetuses examined echocardiographically between June 2016 and August 2020 at the Department of Prenatal Cardiology, Medical University of Lodz, Poland. Only patients who met the inclusion criteria were included in the study. The criteria were as follows: ≥33 weeks of gestation as measured from the first day of mother’s last menstruation (LMP) and a complete medical history including all necessary measurements—namely, IVST obtained by M-mode echocardiography and birth weight (BW). Additional information such as the mother’s age and fetal biometry (EFW and gestational age) were collected from our department’s database.

Prior to ultrasonography and echocardiography, signed written consent was obtained and it was explained to patients that the data might be used for research purposes. Such a policy is approved by the institutional Ethical Committee.

### 2.2. Measurement Tools

**Figure 2:**
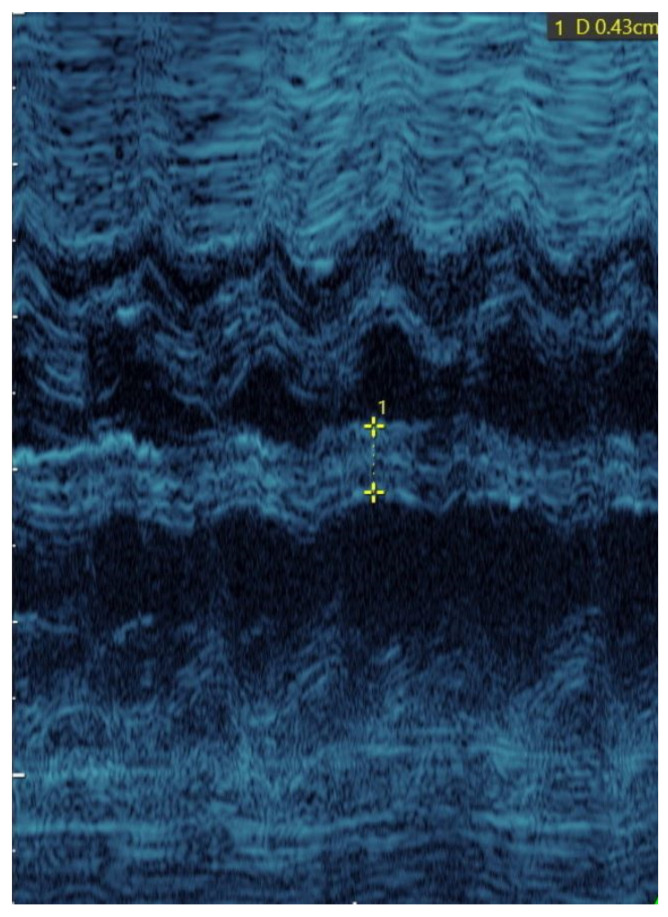
Example of interventricular septal thickness measurement obtained by M-mode echocardiography in the diastole.

Ultrasound was performed at ≥33 weeks of gestation according to LMP. IVST measurements were obtained by M-mode echocardiography in the diastole. The measurements were taken perpendicular to the septum in the middle of its length by a prenatal cardiologist experienced in prenatal ultrasound (Figure 2). IVST was measured 3 times and the average value was calculated to be used in further analyses. BW was measured using an appropriate baby scale approved for professional medical use. An absolute birth weight of over 4000 g was considered the threshold for fetal macrosomia, regardless of gestational age.

### 2.3. Data Collection

We analyzed 3313 fetal echocardiograms obtained between June 2016 and August 2020 at the Department of Prenatal Cardiology, Medical University of Lodz, Poland. 299 patients met the inclusion criteria listed in Section 2.1.

### 2.4. Statistical Analysis

The Statistica 13.1 PL software (StatSoft, Tulsa, OK, USA) was used to generate the receiver operating curve and area under the curve (AUC) to assess the usability of IVST measurement as a macrosomia predictor. The optimal cut-off point was established using Youden’s J statistics [12]. The predictive values—sensitivity, negative predictive value (NPV), specificity, and positive predictive value (PPV)—were calculated using the traditional formulas.

## 3. Results

### Macrosomia Predictors

**Figure 3:**
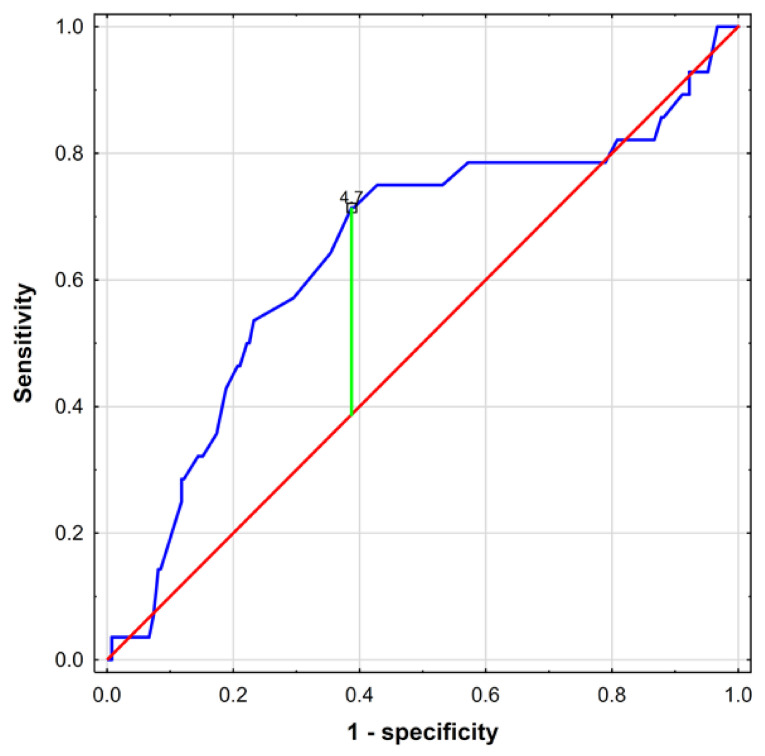
Receiver operating curve for interventricular septal thickness as a novel macrosomia predictor. A cut-off point of 4.7 mm is marked on the curve.

A total of 28 (9.6%) fetuses presented macrosomia, and only 13 (46.43%) of these cases could be predicted based on fetal biometry abnormalities. As is shown in Figure 3, IVST measurement enables the detection of up to 71.43% of macrosomia cases. IVST is therefore a promising macrosomia predictor, with the optimal cut-off point of 4.7 mm (AUC = 0.644; 95% CI: 0.525–0.762; *p* = 0.0177).

The following statistical performance measures were generated, illustrating its predictive potential: 71.43% sensitivity, 95.40% negative predictive value (NPV), 61.25% specificity, and 16.00% positive predictive value (PPV). Therefore, IVST measurement appears to be superior to sonographically obtained fetal biometry where LGA/hypertrophy can be suspected (Table 1). When combined, the analyzed methods (IVST ≥ 4.7 mm and/or LGA/hypertrophy) offer a further performance improvement, with 78.57% sensitivity, 96.27% NPV, 57.20% specificity, and 15.94% PPV (Table 1).

## 4. Discussion

To the best of our knowledge, this is the first study assessing IVST as a potential diagnostic marker of *fetal macrosomia.* It is a common condition affecting approximately 10% of fetuses and potentially leading to serious complications in both mother and child [1,8,9,13]. The resulting complications highlight the need for early diagnosis and prevention [1]. Unfortunately, current predictors such as fetal biometry, fundal height, and amniotic fluid index appear to be insufficient [8,9].

In the search for improved diagnostic tests, serum biomarkers have been studied extensively. Examples of such include microRNA, in particular miR-21, and hormonal biomarkers—namely, adiponectin and insulin-like growth factor-1. Serum metabolites such as glucose, 1,5-anhydroglucitol, and glycosylated hemoglobin have also been shown to facilitate *fetal macrosomia* detection [14,15]. Placental lactogen, which plays a key role in fetal and placental development, is another one of the proposed macrosomia biomarkers [16]. The clinical use of macrosomia serum biomarkers is limited in the era of ultrasonography [16].

Prenatal ultrasound imaging was another subject of research. Ye et al. aimed to investigate whether using ensemble methods on ultrasound measurements could improve the prediction of fetal macrosomia [17]. They concluded that ensemble learning, especially voting and stacking, can improve the prediction of fetal macrosomia and, as such, has the potential to assist obstetricians in making clinical decisions [17].

In our study, we aimed to find a new parameter rather than optimizing existing algorithms. Based on our experience as prenatal cardiologists, we have selected the IVST at ≥33 weeks of gestation as the most promising parameter. The 33 weeks of gestation cut-off point was selected based on our previous experience with diabetic pregnancy, where fetal echocardiography is performed at midgestation to rule out congenital heart defects and, in case of normal heart anatomy, another examination is scheduled for ≥33 weeks of gestation to rule out fetal diabetic cardiomyopathy [10]. This approach allows us to obtain reliable data, since the changes in IVST after 33 weeks of gestation seem to be marginal [11]. Moreover, IVST measurements were taken in diastole, which further improves the reliability of our results by minimizing the effects that certain coexisting cardiac abnormalities may have on IVST measurements. For example, in the case of the volume overload of one of the ventricles, systolic measurements may not be accurate due to pathological IVS movement.

The results obtained in this study confirmed our assumptions that IVST at ≥4.7 mm appears to have a higher sensitivity and NPV than ultrasound, which has been reported both here (Table 1) and elsewhere [1]. Interestingly, a meta-analysis of 29 studies reported ultrasound sensitivity to be 56% [18]. High sensitivity and NPV suggest that IVST measurement offers effective *macrosomia* screening and may be more useful than the current predictors [8,9].

To date, other radiological candidate predictors have emerged—i.e., MRI EFW, with a sensitivity and specificity of 93% and 95%, respectively [18]. Although these values seem to be promising, this approach is considerably more time- and cost-consuming. Consequently, it cannot be used routinely. 

## 5. Strengths and Limitations

This is one of the very first studies focusing on *fetal macrosomia* markers that could be found among parameters routinely obtained by prenatal cardiologists. The study was performed on a representative group (~10% of macrosomia). All the data were obtained by an experienced prenatal cardiologist employed at our department [19]. There was no interobserver variability assessment, as all the measurements were performed by the same prenatal cardiologist. Moreover, we did not consider genetic factors that could potentially affect the fetal/newborn weight [20].

In this study, we are trying to verify suspected abnormalities detected by obstetric ultrasound screening or biochemical tests. Testing a high-risk population can lead to selection bias; however, the prevalence of macrosomia in our study group is in line with the populational prevalence of 10%, as supported by available studies. Detecting a suspected abnormality prior to performing IVST assessment by M-mode echocardiography was necessary, as such examination requires specialized skills and cannot be offered to all pregnant women.

Our results, however promising, require further validation by a prospective study with an even larger study group to enable a more detailed analysis of other macrosomia risk factors.

## Figures and Tables

**Table 1:** Statistical performance measures for interventricular septal thickness at ≥4.7 mm, large for gestational age/hypertrophy on ultrasound, and at least two of these parameters combined. IVST—interventricular septal thickness; LGA—large for gestational age.

	Proposed Tests	IVST ≥ 4.7 mm	LGA in US Exam	IVST ≥ 4.7 mm and/or LGA in US Exam
Predictive Values							
Sensitivity		71.43%	46.43%	78.57%
Specificity		61.25%	77.12%	57.20%
Negative predictive value		95.40%	93.30%	96.27%
Positive predictive value		16.00%	17.33%	15.94%

## Data Availability

Data are available upon request, please contact Iwona Strzelecka (iwona.strzelecka@umed.lodz.pl).
